# Reducing Daytime Sedation While Preserving Motor Function in Parkinson’s Disease Psychosis: A Case Report of Psychotropic Timing and Deprescribing

**DOI:** 10.7759/cureus.114914

**Published:** 2026-08-21

**Authors:** Garrison Burky

**Affiliations:** 1 Psychiatry, Summa Health, Akron, USA

**Keywords:** deprescribing, parkinson's disease psychosis, pimavanserin, psychotropic polypharmacy, quetiapine, sedation

## Abstract

Psychosis and behavioral dysregulation in advanced Parkinson’s disease (PD) are commonly managed with psychotropic medications that may worsen motor symptoms or contribute to excessive daytime sedation. We describe a 77-year-old male with advanced PD and psychosis admitted following agitation-related self-removal of a percutaneous endoscopic gastrostomy (PEG) tube. Collateral history revealed persistent morning somnolence that limited participation in physical therapy and daily care activities. His medication regimen included multiple agents with sedating properties, raising concern for cumulative pharmacologic burden contributing to impaired daytime function. A structured approach to medication adjustments was implemented, focusing on selective deprescribing, consolidating sedating medications to evening dosing, and simplifying the overall regimen. Following iterative medication adjustments, agitation improved without escalation of standing psychotropic medications. Morning alertness improved, with increased participation in therapy sessions, while motor function remained stable. This case highlights an individualized approach to balancing behavioral control, daytime alertness, and motor preservation in PD psychosis while illustrating how cumulative sedative burden and medication timing may influence functional outcomes.

## Introduction

Psychosis occurs in a substantial proportion of patients with Parkinson's disease (PD) and is associated with increased morbidity, caregiver burden, and institutionalization [1-3]. Management is clinically challenging because dopaminergic therapies may exacerbate psychosis, while antipsychotics and other psychotropics may worsen Parkinsonism, cause orthostasis, or produce sedation [2-4]. Complex medication regimens are common in advanced PD, while polypharmacy in older adults increases the risk of medication-related adverse effects and functional impairment [5,6]. In this context, medication timing and cumulative sedative burden may influence clinical outcomes alongside medication selection. Circadian rhythm disruption has been described in PD and may contribute to variability in motor and non-motor symptoms [7,8]. In this patient, these disturbances may have compounded medication-related daytime somnolence and impaired participation in care. PD psychosis commonly includes hallucinations and delusions and may require treatment despite the risk of worsening sedation or motor symptoms [2]. Pimavanserin is a selective serotonin 5-HT2A receptor inverse agonist approved specifically for PD psychosis that avoids direct dopamine receptor blockade [4]. This case focuses not on the initiation of a novel pharmacologic therapy but on selective deprescribing and medication timing as a hypothesis-generating strategy to reduce daytime functional impairment.

## Case presentation

A 77-year-old male with advanced PD complicated by psychosis and behavioral disturbance was admitted from a skilled nursing facility after self-removal of a percutaneous endoscopic gastrostomy (PEG) tube during an episode of agitation. His recent medical course was notable for prior respiratory failure secondary to aspiration pneumonia requiring tracheostomy and PEG tube placement, as well as treatment for an Enterobacter bloodstream infection. He had been undergoing rehabilitation before the agitation episode. Morning somnolence was identified through collateral reports from the patient's spouse and skilled nursing facility staff on admission and was subsequently corroborated through serial observations by the psychiatric team, nursing staff, and rehabilitation therapists during hospitalization. No validated sedation scale was administered. He was frequently unable to participate meaningfully due to decreased alertness. Episodes of agitation occurred intermittently but were described as less functionally impairing than the daily pattern of sedation.

On admission, the patient was clinically stable without evidence of active infection. He was afebrile, with no leukocytosis, and electrolytes were within normal limits (sodium 145 mmol/L, potassium 4.0 mmol/L, magnesium 2.0 mg/dL, calcium 9.0 mg/dL). Renal function was preserved (creatinine 0.7 mg/dL). Mild dehydration was noted, and home furosemide was held. A temporary feeding tube was placed in the emergency department to allow medication administration pending PEG replacement, which was performed on hospital day 2.

His medication regimen included pimavanserin 34 mg daily; quetiapine 25 mg each morning, 125 mg midday, and 150 mg nightly; mirtazapine 7.5 mg nightly; buspirone 5 mg twice daily; benztropine 0.5 mg daily; diazepam 2.5 mg nightly; amantadine 100 mg twice daily; carbidopa/levodopa 25/100, two tablets three times daily; and prazosin 2 mg nightly. Pramipexole 1.5 mg twice daily was documented on admission, but initially could not be administered via PEG. Following replacement of the gastrostomy tube by interventional radiology, dopaminergic therapy was resumed. Pimavanserin 34 mg daily was continued throughout hospitalization as disease-specific therapy for PD psychosis. Psychiatric management focused on sequential modification of quetiapine dosing and reduction of cumulative daytime sedative burden rather than discontinuation of pimavanserin.

**Figure 1:**
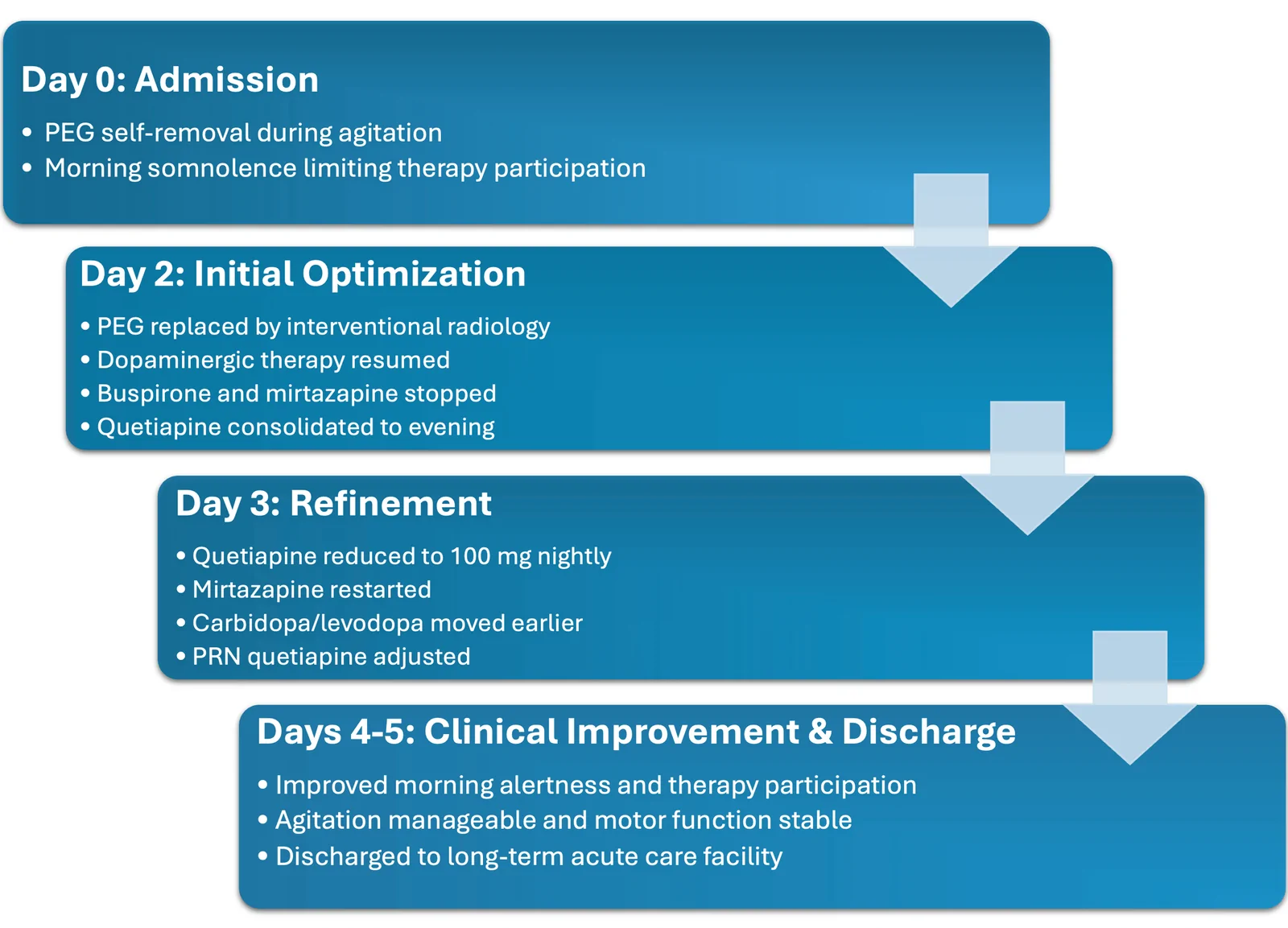
Hospitalization timeline of key clinical events and medication adjustments PEG: percutaneous endoscopic gastrostomy; PRN: as needed

**Figure 2:**
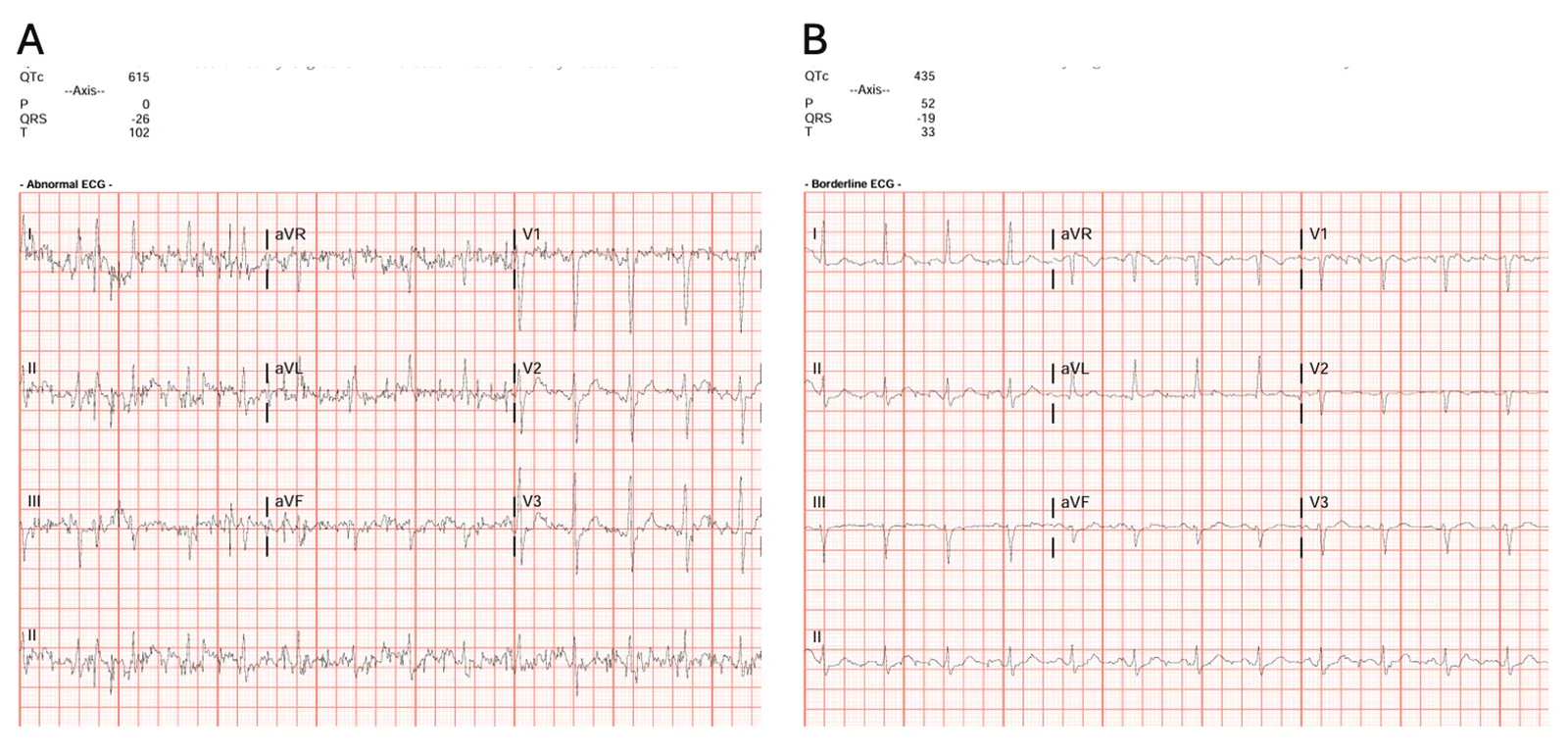
Initial and repeat electrocardiographic findings during hospitalization (A) Initial electrocardiogram with substantial artifact and a machine-reported corrected QT interval (QTc) of 615 ms. Subsequent clinical review determined that the apparent atrial fibrillation represented artifact and that the underlying rhythm was normal sinus rhythm. (B) Repeat electrocardiogram obtained approximately six hours later demonstrating normal sinus rhythm with a machine-reported QTc of 435 ms.

Notably, a substantial portion of quetiapine dosing was administered during daytime hours, contributing to cumulative daytime sedative burden. Multiple agents in this regimen carried sedating properties, including quetiapine, mirtazapine, diazepam, and benztropine [5,6]. Certain medications were continued despite known risks because their anticipated clinical benefits outweighed the risks of immediate discontinuation. Initial electrocardiography reported a QTc interval of 615 ms and was interpreted as atrial fibrillation with rapid ventricular response. Subsequent review determined that the apparent atrial fibrillation represented an artifact and that the patient was in normal sinus rhythm. Repeat electrocardiography approximately six hours later demonstrated normal sinus rhythm with normalization of the QTc to 435 ms. The transient initial finding, therefore, did not prompt a change in the psychiatric medication strategy. The medication regimens before and after the intervention are summarized in Table 1, the hospital course and medication adjustments are illustrated in Figure 1, and the initial and repeat electrocardiographic findings are shown in Figure 2.

**Table 1:** Summary of medication changes and clinical rationale during hospitalization PEG: percutaneous endoscopic gastrostomy

Clinical Action	Medication	Rationale
Discontinued	Buspirone	Limited observed benefit; removed to simplify the regimen.
	Mirtazapine	Initially discontinued because sedation and appetite stimulation outweighed benefit; later restarted at bedtime.
Dose/Timing Modified	Quetiapine	Retimed to bedtime and later dose-reduced to minimize daytime sedation.
	Carbidopa/levodopa	Administration shifted earlier to reduce evening activation.
Temporarily Held	Pramipexole	Held while PEG unavailable; resumed after replacement.
Continued	Pimavanserin	Continued as disease-specific therapy for Parkinson's disease psychosis.
	Diazepam	Continued because family reported benefit; abrupt discontinuation avoided.
	Benztropine	Continued because tremor benefit outweighed immediate deprescribing.
	Amantadine	Continued; no clear acute adverse effects identified.
	Prazosin	Continued because benefit outweighed observed risk.

The initial psychiatric consultation occurred on hospital day 2. The clinical objective was to reduce daytime sedation while maintaining behavioral stability and preserving motor function. Buspirone and mirtazapine were discontinued. Quetiapine was consolidated to 300 mg in the evening (~6 PM), with quetiapine 25 mg twice daily added as needed. Benztropine, prazosin, amantadine, pimavanserin, carbidopa/levodopa, and diazepam were continued. On hospital day 3, the patient was re-evaluated with family present. Overnight, he experienced agitation associated with a nightmare. Scheduled quetiapine was reduced from 300 mg nightly to 100 mg nightly; mirtazapine 7.5 mg nightly was restarted; and carbidopa/levodopa dosing was advanced. Quetiapine as-needed dosing was later increased to 50 mg twice daily based on clinical response.

During the remainder of the five-day hospitalization, agitation was assessed through routine clinical and nursing observation and remained manageable without escalation of standing psychotropic medications. As needed (PRN), quetiapine was used intermittently. Morning somnolence decreased based on nursing and therapy documentation, with increased engagement during scheduled morning therapy sessions compared with admission. Motor function remained clinically stable without documented worsening of parkinsonian symptoms. Standardized measures of agitation, sedation, or motor function were not obtained.

## Discussion

Management in this case required balancing sedation-related functional impairment against the need for behavioral control in the context of advanced PD and complex polypharmacy. The patient’s course occurred in the context of recent medical illness; however, at the time of admission, he was clinically stable without evidence of active infection, electrolyte disturbance, or hemodynamic instability, reducing the likelihood that these factors were the primary drivers of his altered mental status.

A key observation was that a substantial portion of sedating medication exposure occurred during daytime hours. Quetiapine, administered in divided doses with substantial daytime exposure, may have contributed to impaired alertness. In combination with other sedating agents, including mirtazapine, diazepam, and benztropine, this created a cumulative pharmacologic burden not attributable to a single medication but to overlapping effects. Buspirone was discontinued due to limited observed benefit. Mirtazapine was initially discontinued due to its sedating properties and contribution to appetite stimulation in the setting of impaired oral intake, but was later reintroduced at a low nightly dose to support sleep and behavioral stability, reflecting the need for iterative management.

A key intervention was the redistribution of quetiapine dosing to the evening. This approach prioritized reducing daytime receptor exposure while preserving behavioral control. Subsequent reduction in total nightly dose was required after emergence of agitation, raising concern for paradoxical effects in the setting of advanced neurodegenerative disease. Several medications with known risks were continued due to reported benefit and lack of clear alternatives. Diazepam was maintained for nighttime agitation despite known risks in older adults [9]. Benztropine was continued due to reported benefit for tremor. Pimavanserin and carbidopa/levodopa were continued, given their roles in managing psychosis and motor symptoms, respectively. The initially reported QTc prolongation was not sustained; repeat electrocardiography approximately six hours later demonstrated normal sinus rhythm with a QTc of 435 ms, reducing concern that persistent repolarization abnormality required alteration of the psychotropic regimen. Although several medication changes occurred during hospitalization, management was guided by the patient’s dominant functional limitation rather than indiscriminate deprescribing. Morning somnolence consistently interfered with rehabilitation participation, whereas psychosis remained manageable without escalation of standing antipsychotic therapy. Sequential adjustments, therefore, prioritized preservation of daytime function while maintaining behavioral stability and avoiding clinically significant worsening of parkinsonian motor symptoms. This individualized approach illustrates the importance of aligning psychotropic optimization with patient-specific functional goals.

Several limitations constrain the interpretation of this case. Multiple medication changes occurred over a short hospitalization, preventing attribution of improvement to medication timing or any single intervention. Sedation, agitation, and motor function were assessed clinically rather than with validated rating scales, and no formal sedative-burden or anticholinergic-burden instrument was applied. In addition, follow-up was limited to the five-day hospitalization, and the longer-term durability of the observed improvement is unknown.

This case highlights the importance of evaluating cumulative pharmacologic burden rather than individual medications in isolation. Improvement was achieved not by eliminating all potentially inappropriate medications, but by targeted adjustments that reduced overlap in sedative effects and aligned medication timing with functional needs. Thoughtful medication timing may represent a practical strategy in managing complex neuropsychiatric symptoms, although direct evidence in PD psychosis remains limited. This approach may be most relevant to older patients with PD psychosis and complex polypharmacy in whom daytime sedation is interfering with rehabilitation, mobility, participation in care, or other functional goals while behavioral symptoms remain sufficiently controlled to permit cautious medication restructuring.

## Conclusions

This case highlights the importance of medication timing and cumulative sedative burden in PD psychosis, particularly in the setting of complex polypharmacy. In this patient, substantial daytime exposure to sedating medications may have contributed to impaired alertness and reduced participation in care. In this single patient, functional prioritization, selective deprescribing, and redistribution of sedating medications to evening dosing were temporally associated with improved observed daytime alertness and therapy participation while motor symptoms remained clinically stable. Because multiple medication changes occurred simultaneously and standardized outcome measures were unavailable, the contribution of any individual intervention cannot be determined. These observations are hypothesis-generating and suggest that medication timing may warrant consideration in selected patients whose predominant treatment limitation is medication-related daytime functional impairment.
